# A combination of gefitinib and FOLFOX-4 as first-line treatment in advanced colorectal cancer patients. A GISCAD multicentre phase II study including a biological analysis of EGFR overexpression, amplification and NF-kB activation

**DOI:** 10.1038/sj.bjc.6604121

**Published:** 2007-12-04

**Authors:** S Cascinu, R Berardi, S Salvagni, G D Beretta, V Catalano, F Pucci, A Sobrero, P Tagliaferri, R Labianca, M Scartozzi, F Crocicchio, E Mari, A Ardizzoni

**Affiliations:** 1 Department of Medical Oncology, Università Politecnica delle Marche, Ancona, Italy; 2 Department of Medical Oncology, Azienda Ospedaliero-Universitaria di Parma, Italy; 3 Department of Medical Oncology, Azienda Ospedaliera di Bergamo, Bergamo, Italy; 4 Department of Medical Oncology, Azienda Ospedaliera S Salvatore, Pesaro, Italy; 5 Department of Medical Oncology, Azienda Ospedaliera S Martino, Genova, Italy; 6 Department of Medical Oncology, Università della Magna Grecia, Catanzaro, Italy; 7 Research and Development, AstraZeneca, Basiglio, Italy

**Keywords:** colorectal cancer, gefitinib, NF-kB

## Abstract

Interesting activity has been reported by combining chemotherapy with cetuximab. An alternative approach for blocking EGFR function has been the development of small-molecule inhibitors of tyrosine kinase domain such as gefitinib. We designed a multicentre phase II study in advanced colorectal cancer combining gefitinib+FOLFOX in order to determine the activity and to relate EGFR expression and gene amplification and NF-kB activation to therapeutic results. Patients received FOLFOX-4 regimen plus gefitinib as first-line treatment. Tumour samples were analysed for EGFR protein expression by immunohistochemical analysis and for EGFR gene amplification by fluorescence *in situ* hybridisation (FISH), chromogenic *in situ* hybridisation (CISH) and NF-kB activation. Forty-three patients were enrolled into this study; 15 patients experienced a partial response (response rate=34.9%), whereas other 12 (27.9%) had a stable disease. Median progression-free survival (PFS) was 7.8 months and median overall survival (OS) was 13.9 months. We did not find any relationship with EGFR overexpression, gene amplification, while NF-kB activation was associated with a resistance to therapy. Gefitinib does not seem to increase the activity of FOLFOX in advanced colorectal cancer even in patients overexpressing EGFR or with EGFR amplification. Furthermore, while NF-kB activation seems to predict resistance to chemotherapy as demonstrated ‘*in vitro*’ models, gefitinib does not overcome this mechanism of resistance, as reported for cetuximab.

FOLFOX is a generally recognised first-line chemotherapy for metastatic colorectal cancer. Patients receiving this combination chemotherapy achieved a 50% response rate, with a time to progression (TTP) of 9 months and an overall survival of 14 months (de Gramont *et al*, 2000; Goldberg *et al*, 2004; Colucci *et al*, 2005; Meyerhardt and Mayer, 2005).

Recently, interesting activity has been reported by combining FOLFOX or FOLFIRI with EGFR inhibitors. Epidermal growth factor receptor has been demonstrated to be overexpressed in about 70–80% of colorectal tumours and its overexpression is associated with a worse prognosis (Laskin and Sandler, 2004).

Epidermal growth factor receptor inhibition may be obtained by blocking the extracellular part of the receptor (Ellis and Hoff, 2004). Cetuximab, a monoclonal antibody, has been demonstrated to produce a 10% response rate in monotherapy and a 20% when combined with irinotecan even in patients refractory to this drug (Cunningham *et al*, 2004; Lenz *et al*, 2006).

An alternative approach for blocking EGFR function in cancer cells has been the development of small molecules able to interfere with the enzymatic activity of the ligand-activated EGFR (Ellis and Hoff, 2004). Gefitinib is a potent small-molecule inhibitor of tyrosine kinase domain of EGFR. It has demonstrated activity in non-small-cell lung cancer (Cohen *et al*, 2004). It is orally available and is an attractive therapeutic option in colorectal cancer patients. In fact, preclinical data demonstrated a synergism between gefitinib and oxaliplatin and thymidilate synthetase inhibitors (Ciardiello *et al*, 2000; Xu *et al*, 2003; Van Schaeybroeck *et al*, 2005).

Phase I studies showed the feasibility and the safety of the combination of gefitinib with FOLFOX (Kindler *et al*, 2005).

Based on these premises, we initiated a multicentre phase II study in advanced colorectal cancer, combining gefitinib with FOLFOX in order to determine the activity of such a combination and to relate EGFR expression and gene amplification to therapeutic results, as well as if resistance to chemotherapy induced by NF-kB activation may be overcome by gefitinib, as it happens for cetuximab.

## PATIENTS AND METHODS

Patients were considered eligible for this study if they were older than 18 years of age and had histologically confirmed metastatic colorectal adenocarcinoma. Patients were not allowed to receive any kind of prior treatment for their metastatic disease. Other eligibility criteria included measurable disease by Response Evaluation Criteria in Solid Tumors Group (RECIST) criteria, no prior exposition to EGFR inhibitors, an Eastern Cooperative Oncology Group (ECOG) performance status 0–1, adequate blood, renal and liver function. Analysis of tumour EGFR status was not required for inclusion in this study. The treatment protocol was approved by local Ethical Committees. This study was sponsored by AstraZeneca (study number 1839IL/0119).

### Treatment

Initially a cohort of five patients received standard doses of FOLFOX-6 with oxaliplatin at the dose of 100 mg m^−2^ in combination with 250 mg of gefitinib daily. After five patients were treated initially, we decided to expand the cohort to 42 patients, administering FOLFOX-4 at dosages previously published (oxaliplatin 85 mg m^−2^) (Andre *et al*, 1999) due to a better profile of toxicity and tolerability.

Gefitinib at the dose of 250 mg has been taken once a day, every day about the same time. It could be taken with or without food. If the patients forgot to take a dose, they took the last missed dose as soon as they remembered, as long as it was at least 12 h before the next dose is due.

All toxicities were graded according to the NCI-CTC version 2.0. Retreatment at the start of each cycle required adequate haematologic function and resolution of all toxicities of CTC grade 2 or more.

Treatment was continued until development of progressive disease or unacceptable toxicity, withdrawal of patients consent or decision to perform surgical resection of disease.

### Evaluation

Baseline tumour measurements by computed tomography were obtained within 28 days before study treatment was started. Physical examination, including medical history, laboratory studies and assessment of performance status, were conducted at the beginning of each 2-week cycle. Patients were asked to keep a diary of daily gefitinib ingestion and record their experience of nausea and diarrhoea.

Tumour response was evaluated approximately every 8 weeks by computed tomography imaging and tumour measurement performed using RECIST criteria.

### Statistical analysis

Fleming's method was used to calculate the number of patients required (O'Brien and Fleming, 1979).

A sample size of 42 patients is sufficient to give an 80% probability of rejecting a baseline response rate of 50% with an exact 5% one-sided significance test when the true response is at the clinically relevant rate of 70%. The hypothesis that the response rate is equal or less than the baseline is rejected if 27 or more responses are observed out of the 42 patients. Using the methods of A'Hearn (2004), the exact size and power of this test are 4.4 and 83.6%, respectively.

The baseline response rate used for the null hypothesis has been set at 50%. Rejecting the null hypothesis indicates that the activity of the combination is at least similar to that observed using the FOLFOX regimen alone, and therefore that the addition of gefitinib does not compromise the activity of FOLFOX. In the absence of gefitinib, the proposed FOLFOX regimen produces response rates ranging of approximately 50% in the first-line setting, with median progression free survival of 9 months. The addition of gefitinib to FOLFOX is expected to increase the response rate to above 65%. However, the FOLFOX regimen is associated with toxicity, with grade 3–4 neutropaenia observed in 42% of patients, grade 3–4 diarrhoea and grade 3 neurosensory toxicity in 18%. In this study there is a potential for overlapping toxicity, and therefore a higher baseline response rate has not been used to limit the number of patients exposed to the treatment before obtaining further data on safety and some preliminary information on activity.

### Immunohistochemical analysis and gene amplification

Tumour samples, formalin-fixed and paraffin-included, were analysed for EGFR protein expression by immunohistochemical analysis and for EGFR gene amplification by fluorescence *in situ* hybridisation (FISH) and chromogenic *in situ* hybridisation (CISH).

#### Immunohistochemistry

The immunohistochemical study was performed and graded using kit EGFR PharmaDx™ (DakoCytomation, Carpinteria, CA, USA) according to the manufacturer's instructions as previously published (Scartozzi *et al*, 2004).

The EGFR-positive immunostained areas were identified for creation of the section treated with FISH and CISH technique; in addition areas at random were selected in negative tumour for EGFR immunostain.

### FISH analysis of EGFR gene copy number

Analysis of EGFR amplification was performed by using the standard with the dual-colour EGFR Spectrum Orange™/CEP7® Spectrum Green™ probe (Visys, Downers Grove, IL USA). In brief, paraffin sections were deparaffinised, dehydrated in ethanol and air-dried. After treatment in 0.05% pepsin/0.1 N HCl for 45 min at 37°C, the sample were aged in 0.1% NP-40/2 × SSC (standard saline citrate solution) for 10 min at 37°C and their DNA was denatured by treatment in 70% formamide/2 × SSC for 4 min at 85°C. A measure of 5 *μ*l of the probe solution was then placed on a glass slide with a coverslip. The sample slides were hybridised overnight at 37°C and washed, before in 0.4 × SSC/0.3% NP-40 at 73°C for 2 min. Nuclei were counterstained with 4′,6-diamino-2-phenylindole dihydrochloride and *p*-phenylenediamine in phosphate-buffered saline (PBS) and glycerol (DAPI II) (Vysis, Downers Grove, IL, USA). Each FISH assay included normal breast tissue sections, as negative control, and sections of breast cancer previously confirmed to have amplification of EGFR as positive control.

Analyses were performed using a fluorescence microscope (Nikon Optphot-2 and Quips Genetic Workstation) equipped with the Tripple Bandpass Filter set (Vysis) for DAPI II, SpectrumOrange and SpectrumGreen and Filter sets specific to SpectrumOrange and SpectrumGreen.

Only individual and well-delineated cells were scored; overlapping cells were excluded from the analysis. At least 60 cells were scored for each case and control sample.

Each tumour was assessed by the average and the maximum numbers of the copies of EGFR gene per cell, and the average EGFR/chromosome 7 copy number (CEP7) ratio.

Amplification was defined as ratio of EGFR signals to chromosome 7 centromere signals of 2 or more.

### CISH analysis of EGFR gene copy number

Chromogenic *in situ* hybridisation for the EGFR gene was performed according to the manufacturer's instructions (Zymed Laboratories Inc., South San Francisco, CA, USA).

Briefly, the sections of the formalin-fixed and paraffin-embedded tissue were incubated at 55°C overnight. The slides were deparaffinised in xylene and graded ethanols; heat pretreatment was carried out in the pretreatment buffer (Zymed Laboratories Inc.) at 96°C for 15 min.

The tissue was digested with pepsin for 10 min at room temperature, successively was washed with deionised water, dehydrated with graded ethanol and air-dried.

After application of Zymed Spot-Light® oligoxigenin labelled EGFR probe (Zymed Laboratories Inc.), the slides were coverslipped and edges sealed with rubber cement. The slides were heated at 92°C for 5 min, followed by overnight incubation at 37°C using moisturised chamber.

Post-hybridisation wash was performed the next day, followed by immunodetection using the CISH™ polymer detection kit (Zymed Laboratories Inc.).

The CISH signals were seen as dark brown dots and counted in at last 100 nuclei with a light microscope using × 40 objective; only individual and well-delineated cells were scored, and overlapping cells were excluded from the analysis. Also the average gene copies per nucleus for each tissue sections were calculated.

### NF-kB

Nuclear factor-kB was evaluated with an immunohistochemical technique on 3- to 5-*μ*m-thick tissue sections obtained from paraffin-embedded specimens fixed in 10% (v/v) neutral buffered formalin.

The sections were deparaffinised in xylene, rehydrated in graded ethanol, washed in PBS and heated in microwave at 98°C, with EDTA buffer (1 mM; pH 8).

Peroxide blocking was performed with 0.3% H_2_O in methanol at room temperature for 30 min.

The mouse monoclonal antibody raised against amino acids 1–286 of NF-kB p65 of human origin was used (1 : 150 dilution; Santa Cruz Biotechnology, Inc, Santa Cruz, CA, USA) and incubated for 1 h at room temperature.

Incubation with the secondary antibody (EnVision System®; DakoCytomation, Carpinteria, CA, USA/HRP) was performed for 30 min, followed by application of diaminobenzidine chromogen for 5 min. Subsequently, the slides were counterstained with Meyer's haematoxylin for 1 min, dehydrated in a graded series of alcohol, treated with xylene and coverslipped.

The slides were evaluated by light microscopy independently by two pathologists.

Nuclear factor-kB expression was detected as nuclear and cytoplasmic brown staining of neoplastic cell, with various intensity. Positivity of the tumour for NF-kB expression was defined as only distinct nuclear immunostaining, which is considered as activated NF-kB, and is quantified by a percentage score (range 0–100).

The lymphocytes within the tissue sections were used as positive internal controls, which showed positive nuclear staining in all runs.

The negative control was used during optimisation of the method and did not show any staining.

## RESULTS

### Baseline characteristics

Between October 2002 and September 2004, 43 patients were enrolled into this study. Although four patients discontinued chemotherapy before completion of two cycles and were lost to follow-up, all 43 patients enrolled are included in the toxicity and efficacy analyses, on the basis of intention-to-treat.

The baseline characteristics of the 43 patients are listed in Table 1.

### Treatment administration

The mean duration of trial therapy was 26.07 weeks (range 0.14–83.43 weeks).

### Activity

**Figure 1:**
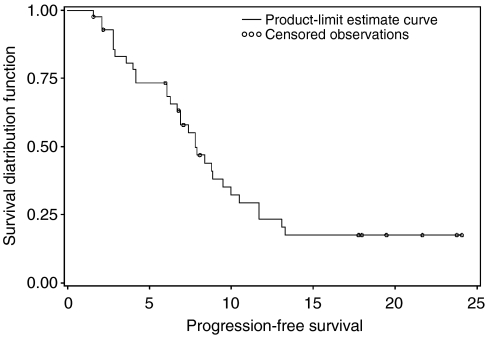
Kaplan–Meier plots for progression free survival.

**Figure 2:**
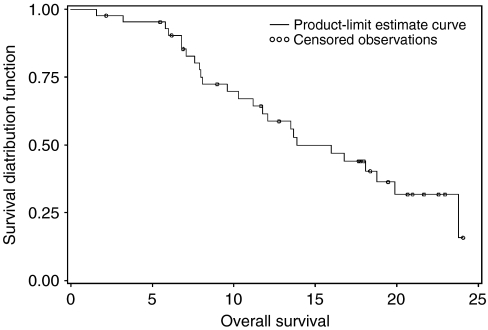
Kaplan–Meier plots for overall survival.

The primary activity end point for this study was objective response rate. By intent-to-treat analysis, 15 patients experienced a partial response (response rate=34.9%), whereas other 12 patients (27.9%) had a stable disease for at least four cycles (Table 2). The secondary efficacy end points were progression-free survival (PFS) and overall survival (OS). The median PFS was 7.8 months (95% CI: 6.7–10 months) (Figure 1). The median OS for all the patients was 13.9 months (95% CI: 11.2–19.9 months) (Figure 2).

### Toxicities

The toxicities of the study regimen represented a secondary end point. Thirty-five out of 43 patients (81.4%) expressed some types of grade 3 or 4 toxicities. Neutropaenia and diarrhoea were the most common side effects. Additional grade 3–4 toxicities included transaminase increase (three patients: in one patient related to gefitinib, in one patient related to FOLFOX and in one patient related to both gefitinib and FOLFOX), leukopaenia related to FOLFOX (three patients), mucositis (two patients: in one patient related to FOLFOX and in one patient related to both gefitinib and FOLFOX) dermatitis or dry skin attributable to gefitinib in one patient and grade 3 peripheral neuropathy attributable to oxaliplatin in one patient. The most common toxicities for all causes are listed in Table 3.

### Immunoistochemistry analysis and gene amplification

Results of EGFR gene analysis by FISH and CISH and NF-kB expression related to clinical response are summarised in Table 4. Data on 20 patients were available for biological analysis. Nuclear factor-kB was activated in four out of five patients who progressed, while it was not activated in all the 10 patients who achieved a PR. The results were statistically significant (*P*<0.05).

Epidermal growth factor receptor was overexpressed in four out of the five patients who progressed, and in six of the 10 patients who had a partial response, but the results were not statistical significant.

Epidermal growth factor receptor was not amplificated in nine out of the 10 patients who achieved a partial response, and in all the five patients who progressed.

## DISCUSSION

The number of available chemotherapy regimens against metastatic colon cancer is rapidly growing. Oxaliplatin or irinotecan in combination with infusional 5FU has been shown to be effective in achieving an improved response and TTP compared with 5FU/LV in colorectal cancer (Grothey *et al*, 2004).

The high level of EGFR response in colorectal cancer specimens has sparked great interest in using this target to develop more direct and specific therapies (Baselga and Arteaga, 2005). To date, positive results with EGFR inhibitors have only been reported for the monoclonal antibody cetuximab. Small-molecule inhibitors of EGFR have recently been tested in combination with chemotherapy in second-line treatment in colorectal cancer. Both gefitinib and erlotinib in combination with FOLFOX or capecitabine and oxaliplatin showed interesting results obtaining higher response rates than those expected with chemotherapy alone. In spite of the lack of benefit for the addition of gefitinib or erlotinib to standard chemotherapy in non-small-cell lung cancer, the results obtained in second-line therapy of colorectal cancer are a strong rationale to move this combination to first line treatment (Kuo *et al*, 2005; Meyerhardt *et al*, 2006).

In this multi-institutional phase II study of the combination of FOLFOX with gefitinib for previously untreated colorectal cancer patients, we observed a response rate of 34.9% and a median PFS of 7.8 months. The median survival for this population of patients was 13.9 months.

Our findings do not compare favourably with similar regimens in first line treatment. In fact, regimens with infusional 5FU and oxaliplatin result in similar response rates and TTP, suggesting that adding gefitinib to FOLFOX does not improve the efficacy of this regimen. These negative results may be due to a lack of efficacy of small molecules inhibitors of EGFR in combination with chemotherapy as in colorectal cancer or to the lack of patient selection (Giaccone *et al*, 2004; Herbst *et al*, 2004; Messersmith *et al*, 2004).

A recent study by Zampino and co-workers also evaluated the combination FOLFOX-6 and gefitinib in a phase II trial including 56 patients. The results showed a higher response (complete response=5.36%; partial response=66.1%) and a higher disease control (91.1%; 95% CI=80.4–97%). Due to the rather small numbers of patients and to the nature of the trial (phase II), in our opinion the differences between the above mentioned and our results are likely due to chance. Nevertheless, a comment could be addressed: in the trial of Zampino and co-workers, patients were enrolled if they had at least 20% cancer cells positive for EGFR, while in our trial only 1% of EGFR positivity was required as per standard practice (Zampino *et al*, 2007).

No biological correlative studies were able to demonstrate a predictive factor for cetuximab therapy activity. Nevertheless, we analysed EGFR overexpression and amplification and NF-kB activation in 20 out of 43 patients receiving gefitinib and chemotherapy. Table 4 summarises these findings. While EGFR overexpression determined by immunohistochemistry is not able to predict response to cetuximab, EGFR amplification was reported to be a good indicator of sensitivity to cetuximab in colorectal cancer. Nuclear factor-kB activation results in an increased resistance to chemotherapy, and the administration of an EGFR inhibitor seems to be able to revert this resistance (Sclabas *et al*, 2003).

In our study, we did not find any relationship between EGFR overexpression and response. In spite of the experience of Moroni *et al* (2005) where about 30% of tumours presented an EGFR amplification, we did not find it in any of our patients.

Similar data were reported by Garufi *et al* (2006) in 70 colorectal cancer patients. In fact, amplification was found in three patients only. Furthermore, Lenz *et al* (2006) reported that EGFR amplification is not related to response to cetuximab, questioning the role of EGFR amplification in the prediction of clinical activity of EGFR inhibitors.

The NF-kB transcriptional factor is constitutively activated in several tumours included colorectal cancer. Furthermore, it is activated by chemotherapy and it represents one of the most important mechanism of cell survival in response to chemotherapy resulting in resistance to treatment (Lind *et al*, 2001; Rakitinat *et al*, 2003; Sclabas *et al*, 2003; Guo *et al*, 2004; Voboril *et al*, 2004; Karin, 2006). In our study, activation of NF-kB was present in four cases. These patients did not respond to treatment, while all the patients with no NF-kB activation presented an objective response. This factor seems to be able to predict resistance to a treatment with FOLFOX and the addition of gefitinib does not overcome this resistance, as some preclinical data suggested for cetuximab (Sclabas *et al*, 2003). Our data are too limited to draw any definitive conclusion; nevertheless in our experience gefitinib does not increase the activity of FOLFOX combination in advanced colorectal cancer even in patients overexpressing EGFR or with EGFR amplification. Furthermore, while NF-kB activation seems to predict resistance to chemotherapy as demonstrated in ‘*in vitro’* models, in our trial, although the small numbers, gefitinib does not overcome this mechanism of resistance as reported for cetuximab.

## Figures and Tables

**Table 1:** Baseline characteristics of patients

**Characteristics**	***n***
No. of patients	43
	
Median age, years (range)	60 (35–75)
	
*Sex*	
Male	23 (53.5%)
Female	20 (46.5%)
	
*Performance status*	
0	30 (69.8%)
1	13 (30.2%)
	
*Received prior adjuvant therapy*	
Yes	9 (20.9%)
No	34 (79.1%)
	
*Metastatic sites*	
1 site	21 (48.8%)
>1 site	22 (51.2%)

**Table 2:** Activity of the treatment

**Parameter**	***n* (%)**
Partial response	15/43 (34.9%)
Stable disease	12/43 (27.9%)
Progressive disease	12/43 (27.9%)
	
Median response duration	5.3 months (range: 0.9–20.9)
Median PFS	7.8 months (95% CI: 6.7–10)
Median OS	13.9 months (95% CI: 11.2–19.9)

CI=confidence interval; OS=overall survival; PFS=progression-free survival.

**Table 3:** Grade 3–4 toxicities (all causes)

**Toxicity**	**All grades (%)**	**Grades 3–4 (%)**
Neutropaenia	26 (60)	18 (42)
Thrombocytopaenia	14 (33)	—
Diarrhoea	28 (65)	13 (30)
Mucositis	10 (23)	2 (5)
Leukopaenia	9 (21)	3 (7)
Transaminase increase	8 (19)	3 (7)
Neuropathy	11 (25)	1 (2.3)
Dermatitis	16 (37)	1 (2.3)

**Table 4:** Available biological data on 20 patients

**NF-kB activation**			
	Yes (4)	No (16)	
PR	0	10	*P*<0.05
SD	—	5	*P*<0.05
PD	4	1	*P*<0.05
			
*EGFR overexpression*			
	Yes (11)	No (9)	
PR	6	4	*P*=NS
SD	1	4	*P*=NS
PD	4	1	*P*=NS
			
*EGFR amplification*			
	Yes (3)	No (17)	
PR	1	9	
SD	2	3	
PD	—	5	

EGFR=epidermal growth factor receptor; NF-kB=nuclear factor-kB; NS=non-significant; PR=partial response; PD=progressive disease; SD=stable disease.
